# High Temperature in Combination with UV Irradiation Enhances Horizontal Transfer of *stx*2 Gene from *E. coli* O157:H7 to Non-Pathogenic *E. coli*

**DOI:** 10.1371/journal.pone.0031308

**Published:** 2012-02-09

**Authors:** Wan-Fu Yue, Min Du, Mei-Jun Zhu

**Affiliations:** 1 Department of Animal Science, University of Wyoming, Laramie, Wyoming, United States of America; 2 Department of Animal Sciences, Washington State University, Pullman, Washington, United States of America; University of Hyderabad, India

## Abstract

**Background:**

Shiga toxin (s*tx*) genes have been transferred to numerous bacteria, one of which is *E. coli* O157:H7. It is a common belief that *stx* gene is transferred by bacteriophages, because *stx* genes are located on lambdoid prophages in the *E. coli* O157:H7 genome. Both *E. coli* O157:H7 and non-pathogenic *E. coli* are highly enriched in cattle feedlots. We hypothesized that strong UV radiation in combination with high temperature accelerates *stx* gene transfer into non-pathogenic *E. coli* in feedlots.

**Methodology/Principal Findings:**

*E. coli* O157:H7 EDL933 strain were subjected to different UV irradiation (0 or 0.5 kJ/m^2^) combination with different temperature (22, 28, 30, 32, and 37°C) treatments, and the activation of lambdoid prophages was analyzed by plaque forming unit while induction of Stx2 prophages was quantified by quantitative real-time PCR. Data showed that lambdoid prophages in *E. coli* O157:H7, including phages carrying *stx*2, were activated under UV radiation, a process enhanced by elevated temperature. Consistently, western blotting analysis indicated that the production of Shiga toxin 2 was also dramatically increased by UV irradiation and high temperature. *In situ* colony hybridization screening indicated that these activated Stx2 prophages were capable of converting laboratory strain of *E. coli* K12 into new Shiga toxigenic *E. coli*, which were further confirmed by PCR and ELISA analysis.

**Conclusions/Significance:**

These data implicate that high environmental temperature in combination with UV irradiation accelerates the spread of *stx* genes through enhancing Stx prophage induction and Stx phage mediated gene transfer. Cattle feedlot sludge are teemed with *E. coli* O157:H7 and non-pathogenic *E. coli*, and is frequently exposed to UV radiation via sunlight, which may contribute to the rapid spread of *stx* gene to non-pathogenic *E. coli* and diversity of shiga toxin producing *E. coli*.

## Introduction

*Escherichia coli* (*E. coli*) O157:H7 and non-O157 Shiga toxin producing *E. coli* (STEC) serotypes, most commonly O26, O111, and O103, are responsible for many food-borne diseases [1] and cause deadly diseases or even death in humans. Recently *E. coli* O104:H4 outbreak in Europe caused thousands of people sick, hundreds of serious hemolytic uremic syndrome and more than thirty death [2]. *E. coli* O157:H7 induces huge losses to the meat industry and is a threat to consumer health [3], [4].

Since the first STEC serotype, *E. coli* O157:H7, was discovered in 1982, more than 500 different serogroups of *E. coli* have been reported to produce Shiga toxin [5]. It is widely regarded that *E. coli* O157:H7 and other STEC obtained the Shiga toxin (*stx*) genes through horizontal gene transfer mediated by lambda phages, because *stx* genes are located in lambdoid prophages within *E. coli* O157:H7 genome [6], [7]. The lambdoid prophages in STEC genomes might be activated similar to lambda phages [7]. UV light activates SOS response, a post-replication DNA repair system, in bacterial cells which enhances the expression of RecA (DNA strand exchange and recombination protein with protease and nuclease activity), leading to the activation of lambda prophages [8]. It is reported that lambda prophage activation requires a threshold temperature of 20°C; below this temperature, lambda prophage cannot be activated, while the higher temperature may enhance its activation [9], [10]. Whether lambdoid prophages in STEC is sensitized similarly by temperature as lambda prophages, and whether there is a synergistic effect of UV light and elevated temperature on its activation has not been tested. However, this is an important question, because global warming increases environmental average temperature, which may accelerate phage activation and the spread of *stx* genes.

Cattle gastrointeninal tract are the natural reservoir of *E. coli* O157:H7. As a result, the sludge in cattle feedlots is densely populated with enteric bacteria including both *E. coli* O157:H7 and non-pathogenic *E. coli* [11], [12]. The extremely high dense population of generic *E. coli* in the feedlot sludge may provide abundant opportunities for the activated *stx* carrying phages to be integrated into the genome of non-toxin producing *E. coli*, generating new STEC. In addition, most feedlots are directly exposed to sunlight which contains a high level of solar UV irradiation. The objective of this study was to test whether high environmental temperature in combination with strong UV radiation provides an environment to generate new STEC, which may provide an explanation for the exploding increase of STEC strains.

## Materials and Methods

### Bacterial strains

*E. coli* O157:H7 strains EDL933, 86-24, 493/89 and 9505 were obtained from the STEC center at Michigan State University. Wild-type laboratory strain, *E. coli* K12 MG1655, was obtained from the American Type Culture Collection (700926™, ATCC, Manassas, VA), which is used as sensitive hosts for induced phages generated by *E. coli* O157:H7 strains. *E. coli* strains were stored in Luria-Bertani (LB) medium containing 15% glycerol at −80°C, which were routinely grown in LB broth at 37°C overnight with aeration.

### Bacterial activation and prophage induction

*E. coli* O157:H7 strains were activated from frozen glycerol stock by culturing in LB media overnight at 37°C with aeration and, then, 1∶100 inoculated into fresh LB medium. The inoculum was cultured at 37°C, 250 rpm till OD_600 nm_ 0.6∼0.8 and, then the culture was divided into two parts. For UV induction, 10 ml of the *E. coli* O157:H7 culture was placed in a 100×15 mm petri dishes with lid off and exposed to UV radiation (0.5 kJ/m^2^) from top. The intensity of UV radiation was measured with a UVX Radiometer equipped with a UVX-25 probe (UVP, Inc., Upland, CA). Following UV radiation, cultures were maintained shaking for 6 h at different temperatures (22, 28, 30, 32, or 37°C). For controls untreated with UV irradiation, cultures were processed exactly the same except UV irradiation. After incubation, culture supernatants were used for analyzing phage titer, Stx2 bacteriophage abundance, and Stx2 protein content.

### Phage enumeration

One ml of *E. coli* O157:H7 culture from different incubation temperature with/without UV radiation was transferred into 1.5 ml micro-centrifuge tubes. The supernatants were collected after centrifuging at 10,000× g for 5 min, and serially diluted with phage buffer containing 10 mM MgSO_4_ and 5 mM CaCl_2_. The appropriate phage suspension dilutions were used for phage forming unit (PFU) analysis. Briefly, 200 µl of phage suspension was mixed with 1 ml of plating host, MG1655 stationary phase culture and 5 ml of tempered top agar (0.7% in 10 mM MgSO_4_ and 10 mM CaCl_2_), and poured onto the bottom LB agar plate per previously published procedure [13], [14], [15]. Plates were incubated at 37°C for 24–48 h and the plaques were counted.

### Quantitative PCR analysis of Stx2 bacteriophages

*E. coli* O157:H7 cultures from different incubation temperature with/without UV radiation were centrifuged (10 min at 10,000× g), and the supernatant was filtered through a sterile 0.22 µm filter (Millipore, Bedford, MA) to completely remove bacteria. One ml of the resulting filtrate was centrifuged at 35,000× g, 4°C for 60 min. The resulting phage pellet was dissolved in sterile ddH_2_O and treated with DNase Ι (2 units/µl) at 37°C for 2 h to digest possible *E. coli* O157:H7 genomic DNA contamination. The resulting phage preparation that only contain packaged phage DNA (phage protein coats protect phage DNA from DNase I) was incubated at 100°C for 10 min to inactivate DNase I and release phage DNA. The prepared phage DNA samples were stored at −20°C and used as a template for quantitative PCR (qPCR) analysis of Stx2 bacteriophages. PCR was conducted using primers specific to *stx2* subunit A (Forward primer: CGTCACTCACTGGTTTCATCAT; Reverse primer: TCTGTATCTGCCTGAAGCGTAA; PCR product size is 133 bp) and SYBR Green master mix (Bio-Rad, Hercules, CA). The specificity of primers was verified by spiking *E. coli* O157:H7 genomic DNA, showing that qPCR signal was directly proportional to the amount of genomic DNA added into the reaction. The amplification efficiency was 0.90–0.99. At the end of each run, dissociation melt curves were obtained to verify that only one PCR product was amplified; the size of amplicon was further confirmed by electrophoresis. Stx2 phage are known to carry only one *stx*2 gene copy, therefore the abundance of *stx*2 gene was extrapolated to the quantity of Stx2 bacteriophage in each sample.

### Western blot analysis of Shiga toxin 2

Immunobloting analysis was conducted according to the procedures described previously [16], [17], [18]. Briefly, protein extractions from the whole bacterial suspension were separated by 10% SDS-PAGE and transferred to nitrocellulose membranes. The transferred nitrocellulose membranes were blocked with 5% milk in PBS, pH7.4 with 0.05% Tween-20, incubated with Stx2A mouse monoclonal antibody (1∶1000 dilution, Toxin Technology Inc., Sarasota, FL) and anti-mouse secondary antibody (Cell Signaling Tech., Boston, MA) consecutively. Blotted membranes were visualized using ECL™ Western blotting detection reagents (Amersham Bioscience, Piscataway, NJ). The density of bands was quantified by using the Quantity One software (Bio-Rad Laboratories, Hercules, CA).

### Horizontal transfer of *stx*2 gene from *E. coli* O157:H7 to non-pathogenic *E. coli*

Wild type laboratory *E. coli* K12 MG1655 was electropolated with the pKNOCK plasmid which carries a Kanamycin resistant gene [19]. MG1655 (Kan^R^) carrying kanamycin resistance was activated from frozen glycerol stock by culturing in LB media overnight at 37°C with aeration. The activated overnight culture was 1∶100 inoculated into fresh LB medium and cultured at 37°C, 250 rpm till OD_600 nm_≅0.6, when 10% (V/V) of the phage preparation obtained from EDL933 was added. MG1655 and phage preparation mixture was incubated at 37°C for 6 h.

### *In situ* colony hybridization to screen newly converted STEC

The newly converted STEC was screened by *in situ* colony hybridization using DIG labeled *stx2* probes. Briefly, the MG1655 (Kan^R^) and phage co-culture prepared above was 10-fold serial diluted and plated to LB with 50 µg/ml Kanamycin (LBKan50) plates. Colonies in plates with 200∼250 colonies were lifted with sterile nitrocellulose (NC) membrane. The NC membrane was inverted and transferred to freshly prepared LBKan50 plates, and incubated at 37°C for 3 h. After incubation, the NC membrane was dried, saturated with 10% SDS, transferred to denaturing solution (0.5 N NaOH, 1.5 M NaCl), neutralizing solution (1.5 M NaCl, 0.5 M Tris Cl pH7.4) and 2× saline-sodium citrate (SSC) washing solution following the published protocols [20]. Then, the membrane was baked at 80°C for 2 h to cross-link DNA to NC membrane and hybridized with DIG label *stx*2 probes synthesized using a PCR DIG Probe Synthesis Kit (Roche, Basel, Switzerland) according to the manufacture's instruction. The positive colonies carrying *stx*2 gene was detected using DIG Nucleic Acid Detection Kit (Roche).

### PCR confirmation of newly converted STEC

*E. coli* O157:H7 EDL933 strain, MG1655 (Kan^R^) and potential positive STEC strains were activated from frozen glycerol stock by culturing in LB media overnight at 37°C with aeration, where EDL933 served as a positive control while MG1655 (Kan^R^) served as a negative control. Overnight culture (200 µl) was centrifuged for 3 min at 10,000× g, the pellet was washed with sterile ddH_2_O twice, then re-suspended in 200 µl sterile ddH_2_O and boiled for 15 min. This crude DNA extract (1 µl) from each strain was used as a template DNA in 25 µl PCR reaction containing 200 µM dNTP, 200 nM each primer and 1 unit of Taq polymerase (NEB, Ipswich, MA). *Stx2* primer sequences (Forward primer: GGCACTGTCTGAAACTGCTCC; Reverse primer: TCGCCAGTTATCTGACATTCTG; Product size = 255 bp) and O157 primer sequences (Forward primer: CGGACATCCATGTGATATGG; Reverse primer: TTGCCTATGTACAGCTAATCC; Product size = 259 bp) were synthesized according to the published sequences [21]. PCR reaction mixtures were electrophoresed on 2% (W/V) agarose gel and stained with ethidium bromide.

### Examination of Stx production by ELISA

Stx production of newly converted STEC was analyzed by ELISA using the ProSpecT Shiga Toxin *E. coli* (STEC) Microplate Assay kit (Thermo Fisher Scientific, Lenexa, KS).

### *E. coli* O157 serotyping

*E. coli* O157:H7, MG1655 (Kan^R^) and potential positive STEC strains were activated from frozen glycerol stock by culturing in LB media overnight at 37°C with aeration. Overnight cultures were boiled at 100°C for 30 min then used for O157 serotyping using an *E. coli* O157:H7 Latex test kit according to the manual instruction (RIM *E. coli* O157:H7 Latex test, Thermo Fisher Scientific).

### Statistics

Data were analyzed using GLM (General Linear Model of Statistical Analysis System, SAS, 2000). Differences in mean values were compared by Tukey multiple comparison, and mean ± standard errors of mean (SEM) is reported. Statistical significance was considered as *P*<0.05. All data are given as means ± SEM of three independent experiments.

## Results

### Rates of lambdoid prophage activation differed among *E. coli* O157:H7 strains

**Figure 1:**
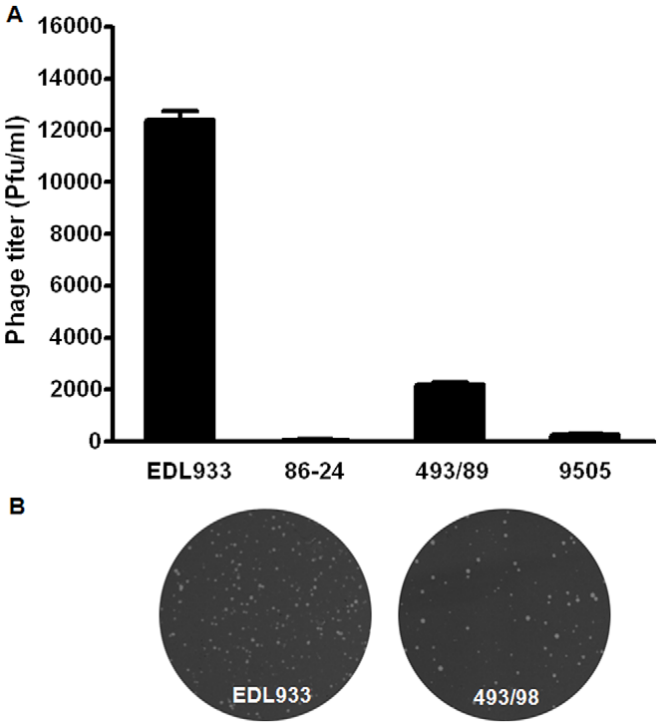
Spontaneous lambdoid prophage induction in *E. coli* O157:H7 strains. (A) Statistical data of prophage induction (Means ± SEM, n = 3). (B) Representative pictures of plaque forming units of *E. coli* O157:H7 strains EDL933 and 493/98.

Four different *E. coli* O157:H7 strains, EDL933, 86-24, 493/89, and 5905, were selected to test the spontaneous activation of lambdoid prophages. As measured by the formation of phage plagues, EDL933 strain had the highest rate of spontaneous activation of prophages, 493/89 stain was the second, and the 86-24 strain had the lowest (Figure 1). Therefore, we chose EDL933 strain for further studies.

### UV radiation and high temperature enhanced activation of lambdoid prophages

**Figure 2:**
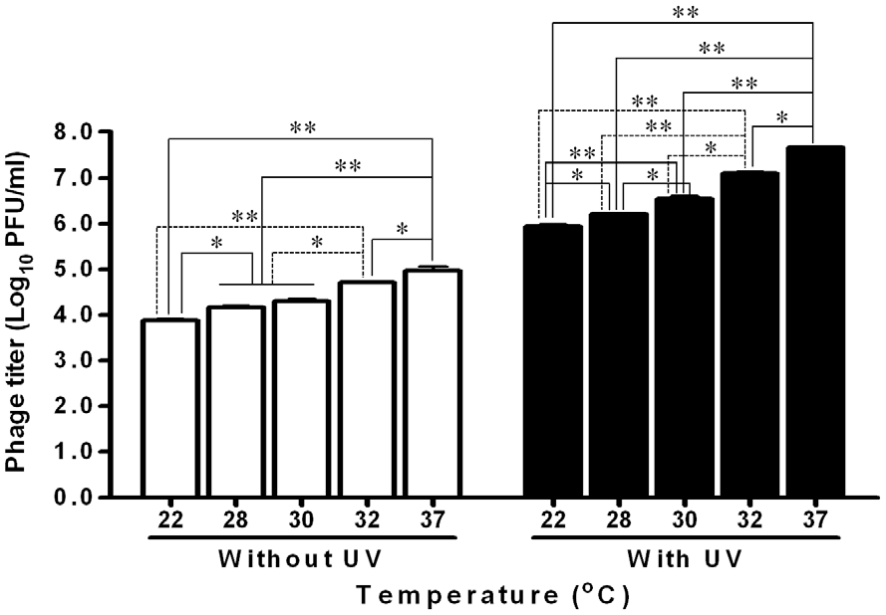
Effects of UV radiation and temperature on lambdoid prophage induction of *E. coli* O157:H7 EDL933 strain. **: *P*<0.01; **: *P*<0.05. (Mean ± SEM; n = 3).

The UV radiation dramatically increased the release of lambdoid prophages (Figure 2). Meanwhile, induction of prophage was affected by temperature (Figure 2). For EDL933 strain, when temperature increased from 22 to 37°C, the inducation of prophages was enhanced (Figure 2). We can also see that temperature and UV irradiation had synergistic effects on lambdoid prophage activation (Figure 2).

### Synergistic effects of temperature and UV irradiation on induction of Stx2 prophages

**Figure 3:**
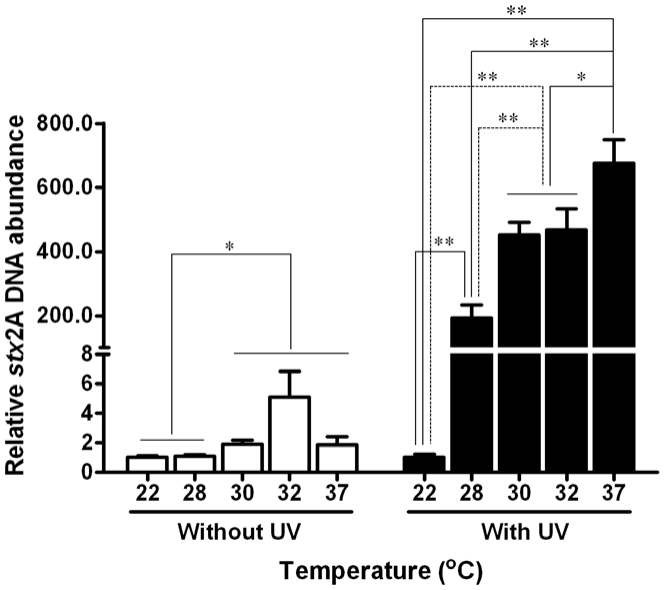
Quantitative PCR quantification of Stx2 prophage induction in the *E. coli* O157:H7 EDL933 strain with/without UV radiation at different temperatures. **: *P*<0.01; **: *P*<0.05. (Mean ± SEM; n = 3).

We further studied the induction of Stx2 prophages under different temperature and UV irradiation combination by quantitative PCR using primers specific to *stx*2 gene subunit A. Since Stx2 phage contains one copy of *stx*2 gene, the abundance of *stx*2 gene subunit A was referred to the quantity of induced Stx2 phages. At all test temperature except 22°C, UV irradiation dramatically enhanced the induction of Stx2 prophages (Figure 3). In non-UV treated control, induction of Stx2 prophage at 28°C was not different from that at 22°C, however, induction of Stx2 prophage was significantly higher at 30, 32 and 37°C compared to that at 22°C. In the UV treated group, induction of Stx2 prophage increased several hundred folds when temperature increased from 22°C to 28, 30, 32 or 37°C (Figure 3); the induction of Stx2 phage was the highest at 37°C, followed by 30 and 32°C (Figure 3). These results indicated that there were synergistic effects between UV irradiation and environmental temperature on Stx2 prophage induction.

### Effects of UV radiation and temperature on Shiga toxin 2 production

**Figure 4:**
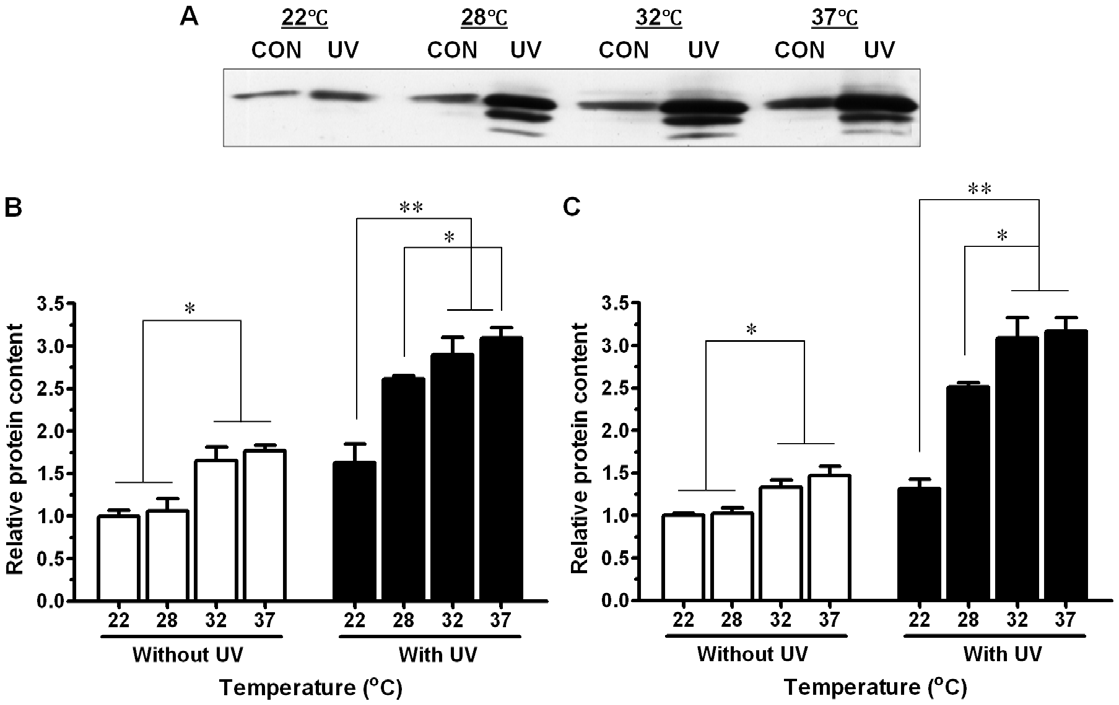
Effects of UV radiation and temperature on Shiga toxin 2 protein content in *E. coli* O157:H7 EDL933 strain. (A) Representative Stx2A western blotting band. (B) Relative Stx2A protein content including only the major band. (C) Relative Stx2A protein content including all three bands. **: *P*<0.01; **: *P*<0.05. (Mean ± SEM; n = 3).

Consistent with the enhanced Stx2 prophages induction, UV treatment and elevated temperature increased the protein content of Stx2 (Figure 4). As for Stx2 prophages induction, there was synergistic effect between UV and temperature on Stx2 production (Figure 4). Interestingly, western blotting analysis detected three bands of Stx2A subunit in samples treated UV irradiation, while only single band (MW = 37 kD) in non-UV treated samples. The reason for these two lower MW bands appeared in the UV treated group is unclear (Figure 4).

### Horizontal gene transfer of *stx*2 gene from Stx2 phage to non-pathogenic *E. coli*

**Figure 5:**
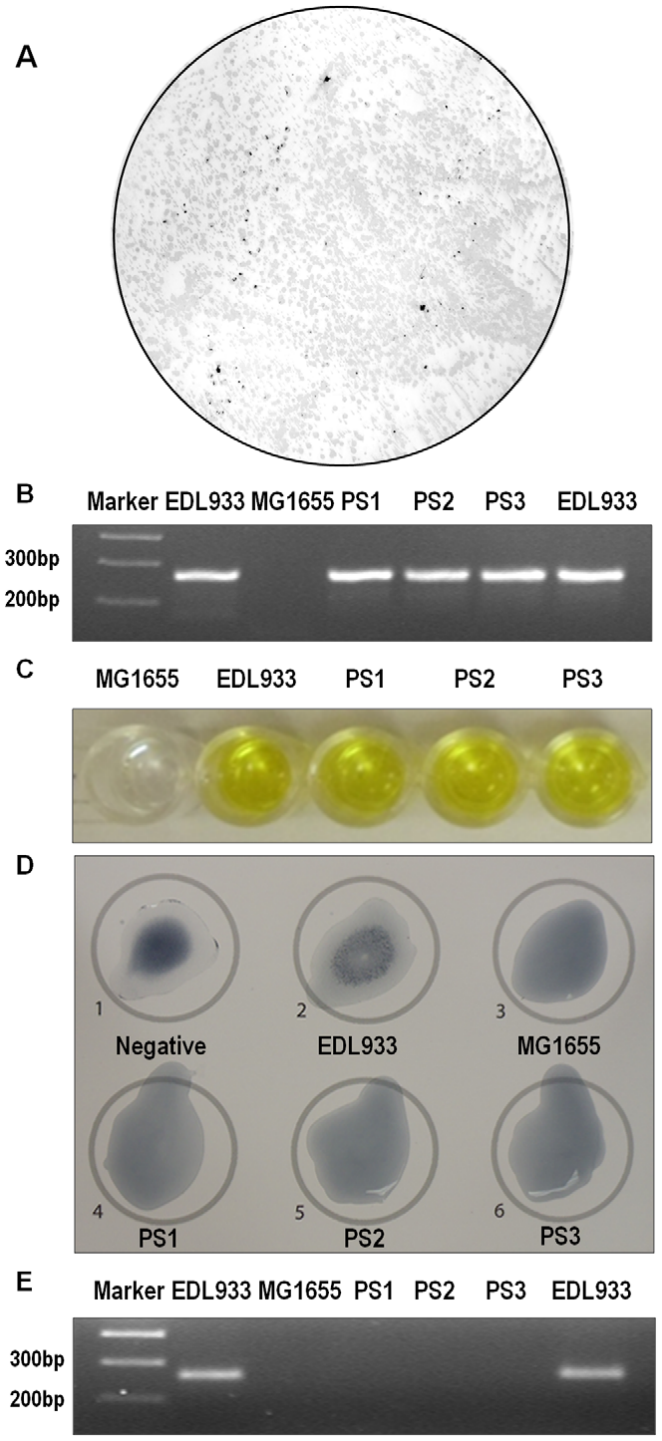
Confirmation tests on newly converted STEC. (A) Representative picture of *in situ* colony hybridization with DIG labeled *stx*2A probe; (B) PCR confirmation of *stx*2A gene in newly converted STEC strains (PS1–PS3); (C) ELISA detection of Shiga toxins; (D) O157 serotyping of newly converted STEC; (E) PCR confirmation of O157 gene was absent in newly converted STEC strains (PS1–PS3).

To test whether induced Stx2 phage has the ability to convert non-pathogenic *E. coli* to STEC, phage preparation following EDL933 induction was incubated with an *E. coli* K12 strain MG1655 (Kan^R^). As shown in Figure 5A, a number of *stx*2 positive colonies were detected by *in situ* colony hybridization. To further confirm, three positive colonies were selected which were designated as PS1–3. PCR analysis indicated that PS1–3 contained *stx*2 gene (Figure 5B). ELISA analysis further confirmed that PS1–3 produced Stxs (Figure 5C). O157 agglutination assay indicated that PS1–3 as well as MG1655 (Kan^R^) were O157 negative. We further analyzed the presence of O157 gene by PCR using primers specific to O157 gene. As indicated in Figure 5E, O157 gene was only detected in *E. coli* O157:H7, but not in MG1665 (Kan^R^) and PS1–3. All these indicated that PS1–3 were newly generated STEC through horizontal gene transfer.

## Discussion

Shiga toxin genes are mainly responsible for the pathogenesis of *E. coli* O157:H7 through Stx-induced renal endothelial injury [22]. *E. coli* O157:H7 and non-O157 STEC serotypes are responsible for many food-borne diseases [1]. Beef product recalls due to *E. coli* O157:H7 contamination cause huge losses to beef industry. Further, the runoffs from cattle farms contaminate vegetables causing additional safety concerns. Therefore, it is important to prevent *E. coli* O157:H7 and other STEC contamination and propagation. Very recently, European toll in the outbreak of a rare STEC strain, *E. coli* O104:H4, in Germany and France had risen to 855 cases of HUS, 2,987 cases of acute gastroenteritis and 35 deaths, highlighting the seriousness of food safety problems associated with STECs [2].

Cattle gastrointestinal tract is the primary reservoir of *E. coli* O157:H7 and non-O157 STEC [23], and fecal shedding of *E. coli* O157:H7 is the major source of this pathogen [24]. The prevalence of *E. coli* O157:H7 in the intestinal tracts of feedlot cattle is alarmingly high, ranging from around 10 to 20% [11], [25]. Due to the high rate of *E. coli* O157:H7 shedding in feces, feedlot sludge is highly contaminated with *E. coli* O157:H7. In addition, other STEC such as members of the O26, O91, O103, O111, O118, O145, and O166 serogroups are increasingly detected in beef feedlots, which also have been isolated from beef and associated human cases [23]. These STECs co-exist with extremely dense populations of enteric bacteria in feces, which accumulate in the feedlot sludge. The abundance of both STEC and non-STEC, in combination with the high moisture content of feedlot sludge, may provide an ideal environment for horizontal transfer of *stx* genes from STEC to non-pathogenic *E. coli*.

Shiga toxin genes likely were captured by STEC through horizontal gene transfer [26]. There are 18 lambdoid prophages identified in the *E. coli* O157:H7 genome. Both *stx*1 and *stx*2 genes locate in lambdoid prophages, with *stx*2 gene in prophage 5 and *stx*1 gene in prophage 15 [6], [7]. Though these prophages are defective in certain genes required for phage induction and propagation individually, a very recent study shows that as a pool, these prophages can compensate each other's defects and generate active phages carrying *stx*2 genes [7]. We tested the spontaneous induction of phages in four different *E. coli* O157:H7 strains, EDL933, 86-24, 493/89, and 5905, and the EDL933 strain had the highest rate of spontaneous induction of prophages while the 86-24 strain had the lowest. The difference in phage activation may be due to the different degree of lambdoid prophage degeneration; high degree of degeneration reduces and abolishes the ability of lambdoid prophages to be activated [27].

It has been demonstrated earlier that lambda prophage activation is temperature dependent; higher temperature decreases the stability of phage CII protein, promoting the lytic cycle of prophages [9]. The lytic pathway is blocked when temperature below 20°C [10]. In this study, similar temperature-dependent induction of lambdoid prophages was observed. In addition, we also observed that lambdoid prophages in *E. coli* O157:H7 were dramatically induced by UV radiation at a level comparable to the radiation of sunlight in western high plains. More importantly, we observed synergistic effects between temperature and UV radiation on lambdoid prophage induction, including *stx*2 prophages.

For the propagation of *stx* gene, those activated lambdoid prophages carrying *stx*2 genes must be integrated into the genome of non-STEC. Indeed, we demonstrated that induced Stx2 prophages were able to convert a non-pathogenic *E. coli* into STEC. In a previous study, lysogenic infection of Stx phages was also detected in a pig farm [28].

In recent decades, climate change characterized by global warming significantly increases environmental temperature. It is expected that temperature will continue to increase during this century. In addition, most cattle feedlots are not or only partially shaded, and exposed to sunlight. High temperature in combination with UV radiation, plus the abundant STEC and other enteric bacteria in cattle sludge, may provide an ideal reservoir for the generation of new STEC from non-STEC. New pathogens, once generated, could rapidly propagate to other regions through cattle transportation, and beef and food distribution, threatening the health of Americans and even globally.

In summary, following the first identification of STEC, *E. coli* O157:H7, in 1982, *stx* genes have spread to more than 500 serogroups of *E. coli*, and other bacteria [5]. If no preventive strategies are taken, *stx* genes are expected to spread further. Our data demonstrate that high temperature and UV radiation comparable to the environment of cattle feedlots cause induction of lambdoid prophages carrying *stx* genes, which can convert non-pathogenic *E. coli* into STECs. Our observation provides a possible explanation for the widely spreading of *stx* genes in the last decades.
